# Menarche in Scoliotic and Non-Scoliotic Balkan Girls and the Relationship between Menarche and the Laterality of Scoliotic Curves

**DOI:** 10.3390/jcm13010132

**Published:** 2023-12-26

**Authors:** Samra Pjanic, Nikola Jevtic, Theodoros B. Grivas

**Affiliations:** 1 Department of Pediatric Rehabilitation, Institute for Physical, Rehabilitation Medicine and Orthopedic Surgery “Dr Miroslav Zotovic”, 78000 Banja Luka, Bosnia and Herzegovina; samra.pjanic@hotmail.com; 2 Scolio Centar, 403916 Novi Sad, Serbia; njevticns@gmail.com; 3 Former Head of Department of Traumatology and Orthopaedics, “Tzaneio” General Hospital of Piraeus, 18536 Piraeus, Greece

**Keywords:** menarche, idiopathic scoliosis, Balkan

## Abstract

Background: Menarche, as an important parameter in the assessment of scoliosis progression in girls, is proven to be dependent on geographical latitude. The aim of this study was to determine whether the age of menarche differs in scoliotic and non-scoliotic Balkan girls and the relationship between menarche and the laterality of scoliotic curves. Participants and Methods: This is a retrospective study with three groups: scoliotic, non-scoliotic, and control. Patient data collection and analysis were approved by the Ethical Committee of the Institute. Statistical Analysis: The SPSS 24 program was used, and we employed One-way ANOVA, Fisher’s, and Chi-squared tests to compare different groups. Statistical significance was defined as *p* < 0.05. Results: No statistically significant difference was found in the age of menarche between the three groups (*p* = 0.168). In the scoliotic postmenarchal group, the primary right curve was dominant in 54.80%, while in the scoliotic premenarchal group, the primary left curve was dominant in 60.09% (*p* < 0.01). Conclusion: In Balkan girls from Bosnia and Herzegovina and Serbia, there was no significant difference in the age of menarche between scoliotic and non-scoliotic girls. A significant difference was found in the laterality of the primary curve in premenarchal and postmenarchal scoliotic girls.

## 1. Introduction

Idiopathic scoliosis represents a three-dimensional deformity of the spine and thorax that can occur at any age in growing children, and it especially progresses during the pubertal rapid growth spurt. IS occurs in 80% of all scoliosis cases, while the remaining 20% represent secondary scoliosis due to other pathological conditions [1]. Depending on the age of occurrence, it can be classified as infantile (0–3 years of age), juvenile (4–9 years of age), and adolescent idiopathic scoliosis (10–18 years of age) [2]. The greatest risk of progression is expected during puberty due to the peak height velocity. Progressive cases of AIS are much more frequent in girls than in boys, with a ratio of 7:1 in favor of girls, with Cobb angle values above 30° [1].

Many studies have been conducted regarding the aetiopathogenesis of IS [3,4], suggesting there is a multifactorial cause including genetic [5] and epigenetic factors [6]; the central nervous system; skeletal spinal growth; bone metabolism; metabolic pathways; biomechanics; and other factors, yet its etiology still remains unclear [7,8].

The risk of scoliosis progression is related to growth potential, which can be determined by the assessment of skeletal maturity. Bone age as an essential parameter for evaluating the remaining growth during puberty can be determined based on pelvis and upper limb radiographs (elbow, hand, wrist and humeral head ossification [9]). One of the most important and used indicators of skeletal maturity is the Risser sign [10], due to the fact it can often be assessed on the same spine radiograph as the Cobb angle. The Risser sign is assessed by the ossification of the iliac apophysis divided into five stages (0 to 5) and is displayed on full AP or PA spine radiographs on the pelvic iliac crest. The most growth potential is expected between Risser sign 0 and 2, whereas Risser sign 0 covers about two-thirds of puberty and therefore can be misleading [11]. Therefore, the Risser sign has been proven to have limited sensitivity during peak growth velocity, and there is the possibility of high mismatch risk and mistreatment [12]. For this reason, there is a need for additional parameters that can help in the assessment of growth potential and risk of scoliosis progression.

There are also some other skeletal maturity indicators, such as Sanders [13] and elbow triradiate cartilage classification [14], that are not widely utilized in clinical practice due to additional radiation (the need to radiograph the wrist or elbow). There is a clear correlation between growth velocity and skeletal maturation [15].

Among the clinical parameters, data about age at menarche are considered to be important, along with biometric measurements and Tanner stages [16]. The most important biometric measurements include standing height, sitting height, arm span, and weight, which should be repeated and recorded at regular intervals. The sitting height is more accurate than the standing height since the trunk and lower extremities grow at different rates. When the sitting height is approximately 84 cm, 80% of girls experience menarche. The Tanner stages signal the development of secondary sexual characteristics throughout the course of puberty. Tanner stage 2 marks the beginning of puberty, with the first appearance of pubic hair, the budding of the nipples, and the swelling of the testes as the first physical signs of the onset of puberty [11]. The age at the menarche onset is also one of the indicators of the remaining growth potential in girls [17]. Some authors state that it is considered to be even more reliable than the Risser sign [18].

The first physical sign of puberty occurs about 2 years before menarche, and final height is usually achieved 2.5–3 years after menarche. In girls, menarche occurs at age 13.5 years of bone age, usually at Risser grade I [11]. After girls experience menarche, there is a gradual decrease in the scoliosis progression risk. This is due to the fact that at the time of menarche, approximately two-thirds of the period of the pubescent growth spurt and the peak of growth have passed. The potential for scoliosis progression is much lower after spinal growth and skeletal maturation are complete [2].

The late onset of menarche correlates with delayed skeletal maturity and a higher prevalence of AIS. For this reason, in girls who experience menarche later, there is a prolonged period of spine vulnerability and a greater possibility of scoliosis progression [19].

Taking everything above into consideration, data about menarche in girls can provide clinicians with additional information about remaining growth, helping them to assess the risk of progression and therefore guide them in treatment decisions.

For this reason, the age of menarche onset is a parameter that has been documented during the clinical assessment and follow-up of scoliotic patients in many clinics around the world and during school screening programs.

There are a few studies showing that melatonin may play a role in the pathogenesis of scoliosis, supporting the neuroendocrine hypothesis [20]. Melatonin, as a hormone produced mainly in the pineal gland but also in the retina, is called “the light of the night” since its synthesis and release are stimulated by the darkness and inhibited by the light. Among many other roles, melatonin influences the sexual maturation process by reducing the secretion of gonadotropins, mainly LH. This is related to the occurrence of menarche, which arises as the melatonin levels decrease during growth, influencing the onset of puberty [21,22,23].

This can explain why different sunlight exposure, depending on geographical location and equatorial distance, influences melatonin secretion and modifies age at menarche. Therefore, the role of melatonin can be extended to the findings that menarche depends on geographical latitude [24,25,26,27,28].

This might be a reason why reported AIS prevalence in the literature increases in the northern geographic latitudes and decreases with latitudes approaching the equator [29,30,31,32,33,34,35,36,37,38,39,40,41,42,43,44,45].

This claim is supported by the epidemiological data on AIS prevalence in different countries, showing that Finland has the highest geographical latitude and an AIS prevalence of 12% and Singapore has the lowest geographical latitude and an AIS prevalence of 0.93%. When assessing the influence of geographic factors, it is important to distinguish them from socioeconomic circumstances [46].

There is also a higher prevalence of scoliosis in a population of blind women (42.3%) compared to the prevalence in the general population of the same region (2.9%) [47]. This finding contributes to the possible role of melatonin in IS etiopathogenesis. In blind women, there is an increased production of melatonin due to a lack of light, which leads to late menarche and a prolonged period of spine vulnerability.

The only country in the Balkan region that has published data on AIS prevalence is Greece [48].

When comparing scoliotic and non-scoliotic girls in the Mediterranean region, no statistically significant difference has been found in the age of menarche. On the other hand, there was a statistically significant difference between menarche-positive and menarche-negative scoliotic girls in relation to the laterality of scoliotic curves [17]. The laterality of the curve is defined as the deviation of the spine in the frontal plane and is represented by the Cobb angle, measured on the full spine radiograph in PA projection. It is the most documented measurement, frequently used in scoliotic patients for follow-up and treatment outcomes, and it is also considered one of the main predictors of progression [49].

To our knowledge, there are no data about age of menarche in scoliotic and non-scoliotic girls in the Balkan countries of Serbia and Bosnia and Herzegovina.

The aims of the study were to determine whether the age of menarche differs in scoliotic and non-scoliotic Balkan girls; to compare the results to the findings from other countries, and to assess the relationship between menarche and the laterality of scoliotic curves.

## 2. Patients and Methods

### 2.1. Study Design

A retrospective study based on prospectively collected clinical data was conducted in Bosnia and Herzegovina and Serbia.

### 2.2. Participants

Participants for the study were selected among 2000 patients of the Institute for Physical, rehabilitation medicine and orthopedic surgery “Dr Miroslav Zotovic” in Banja Luka (Bosnia and Herzegovina) and healthy girls randomly selected during screening in sports clubs in Novi Sad (Serbia). Patient data collection and analysis were approved by the Ethical Committee of the Institute.

### 2.3. Methods

Criteria for the inclusion in the study were gender and Cobb angle. Since our research is based on menarche, boys were automatically excluded from the study. The girls enrolled in the study were referred to our clinic due to clinical trunk asymmetry detected during official school screening or by other health professionals or parents. The girls who showed asymmetry in the Adam’s bending test and had scoliometer readings ≥5° were referred for further diagnostics. The golden standard used to diagnose scoliosis consisted of a full spine radiograph in PA projection performed in our clinic. Scoliosis was defined by the SRS definition as the curve with a Cobb angle ≥10°. Scoliosis was excluded if the Cobb angle was <10°. The Cobb angle was measured in the TraumaCad^®^ (https://www.brainlab.com/surgery-products/orthopedic-surgery-products/orthopedic-templating-software/ (accessed on 23 October 2023)) software program with a focus on the primary scoliotic curve, defined as the curve with the most structural vertebral changes in terms of lateral deviation and rotation. The girls in Serbia were examined as part of the regular screening program in amateur sports clubs, where they were engaged in some type of sports training up to 4 times a week. In the control group, we included the girls with no trunk asymmetry in the standing position or Adam’s bending test or with any signs of scoliosis.

According to the above-described parameters, the participants were divided into three groups: scoliotic group, non-scoliotic group, and control group.

The scoliotic group consisted of 494 patients, with a mean age of 12.57 ± 2.4 years (interval 7 to 18 years) and an average Cobb angle of 20.44 ± 10.88°. The non-scoliotic group consisted of 523 patients, with a mean age of 11.51 ± 2.71 years (interval 7 to 18 years) and an average Cobb angle of 6.71 ± 1.76°. The control group consisted of 86 healthy girls, with a mean age of 13.63 ± 1.54 years (interval 10 to 17 years).

## 3. Statistical Analysis

Statistical analysis was performed using the program SPSS Inc. (Chicago, IL, USA), released in 2016 (SPSS for Windows, Version 24.0. Chicago, IL, USA).

The results are presented as frequency and percentage (for all participants in three main groups—scoliotics, non-scoliotics, and control and subgroups—according to the age of menarche and BMI level), mean and standard deviation (SD) (for average age and body height of the participants), and median and interquartile range—IQR (for Risser sign). One-way ANOVA was used to compare the groups and determine if there were statistically significant differences between them, measured on interval scale, with normal distribution. Tukey’s HSD test was used as a post hoc test if there was significant difference between groups. Fisher’s test was used to compare the groups measured on nominal or ordinal scales (BMI level). A Chi-squared test was used when the frequency in the categories was less than 5. Statistical significance was defined as *p* < 0.05.

## 4. Results

There were 1017 girls selected among patients for the first 2 groups and 86 healthy girls in the control group.

Data on the height of all participants and according to the age of menarche, as well as a comparison between the groups, are presented in Table 1.

There was a significant difference in height between the three groups (*p* < 0.001). The girls in the control group were significantly taller than the participants in the other two groups (scoliotics and non-scoliotics). With post hoc analysis and according to the age of menarche, a significant difference was found in premenarchal girls between the control and non-scoliotic groups (*p* < 0.001), as well as in postmenarchal girls between the control and scoliotic groups (*p* = 0.020).

To form BMI categories for the participants, the CDC growth reference chart for girls was used, with five categories according to percentiles: Underweight (<5 percentiles), Healthy Weight (5 to 85 percentiles), Overweight (85 to 95 percentiles), Obesity (>95 percentiles), Severe Obesity (>95 percentiles × 1.2), Table 2.

There was no statistically significant difference between the scoliotic, non-scoliotic and control groups according to BMI for all girls (*p* = 0.073), premenarchal girls (*p* = 0.560) and postmenarchal girls (*p* = 0.128). There was also no statistically significant difference in the scoliotic group between premenarchal and postmenarchal girls (Chi-squared test, *p* = 0.371).

The Risser sign was homogenized and there was no significant difference between the groups (Chi-square test, *p* = 0.461).

Data about the age of participants and age at menarche in all three groups are presented in Table 3.

*In the scoliotic group*, 281 girls (56.88%) with a mean age of 14.04 ± 1.50 (range 10–18) were postmenarchal (mean age at menarche 12.38 ± 1.11), while 213 girls (43.12%) with a mean age of 10.63 ± 1.95 (range 7–16) were premenarchal.

*In the non-scoliotic group*, 218 girls (41.68%) with a mean age 13.92 ± 1.69 (range 11–18) were postmenarchal (mean age at menarche 12.18 ± 1.22), while 305 girls (58.32%) with a mean age of 9.78 ±1.85 (range 7–15 years) were premenarchal.

*In the control group*, 69 girls (80.23%) with a mean age of 14.07 ± 1.28 (interval 12–17) were postmenarchal (mean age at menarche 12.26 ± 1.22 (range 10–15), while 17 girls were premenarchal (mean age at menarche: 11.82 ± 1.19 (range 10–14).

No statistically significant difference was found in age of menarche between the three groups (*p* = 0.168), presented in Table 4.

The mean Cobb angle of non-scoliotic girls was 6.71 ± 1.76°. In scoliotic girls, the average Cobb angle was 20.44 ± 10.88°; in the postmenarchal scoliotic group, it was 21.89 ± 11.04°, while in the premenarchal scoliotic group, it was 18.43 ± 9.98°.

The mean Cobb angles in postmenarchal and premenarchal scoliotic girls, according to the laterality and localization of the primary scoliotic curve, are presented in Table 5.

The Cobb angle, as a quantitive measure of curve magnitude, was analyzed in relation to menarchial status and the laterality of the curve in scoliotic curves. There was no statistically significant difference in Cobb angle between postmenarchal and premenarchal girls with a right scoliotic curve (t = 0.930, *p* = 0.353). On the contrary, a statistically significant difference was found between postmenarchal and premenarchal girls with a left scoliotic curve (t = 4.297, *p* < 0.001). The results are presented in Table 6.

Furthermore, there was a statistically significant, but very weak, negative correlation between the Cobb angle and menarchial status (r = −0.159, *p* < 0.001), while there was no statistically significant correlation between the Cobb angle and the laterality of the primary scoliotic curve (r = −0.061, *p* = 0.176).

In the scoliotic group of girls, a statistically significant relationship was found between the age at menarche and the laterality of the primary scoliotic curve (χ^2^(1,n = 494) = 10.18, *p* < 0.01, phi = 0.148).

In postmenarchal scoliotic girls, the primary right curve was dominant in 54.80%, while in scoliotic premenarchal girls, the primary left curve was dominant in 60.09% (*p* < 0.01). These results are shown below in Table 7 and Table 8.

## 5. Discussion

Menarche in girls is widely considered to be one of the important parameters in the assessment of the risk of scoliosis progression and one of the influencing factors in the etiopathogenesis of IS. The age of menarche can indicate the remaining growth potential in girls [12] and be even more reliable than a Risser sign [18].

However, the Risser sign as a skeletal maturity indicator has been widely used by professionals in clinical settings. Nonetheless, its accuracy in the assessment of the risk of scoliosis progression has been undermined lately due to reported limitations, especially during growth spurts. It has been shown that there is a high risk of mismatch in patients assessed with Risser staging compared to the Sanders classification [12]. For this reason, Risser staging cannot be used exclusively to assess the risk of progression and remaining growth.

Regardless of its significance, there are only a few studies that highlight the importance of menarche in girls.

In 2002, Grivas et al. [17] answered the pending question of whether there is a difference in the age of menarche between scoliotics and non-scoliotics among Mediterranean girls. They found no statistically significant difference in the age at menarche between scoliotic and non-scoliotic girls. Furthermore, Mediterranean girls were younger in age compared to their counterparts in North Europe. These results are in accordance with the results of our study, which can be explained by similar geographical latitude of the Balkan countries (Greece 38°16′29.82″ N, 22°00′ E, Bosnia and Herzegovina 43.9159° N, 17.6791° E, Serbia 44.0165° N, 21.0059° E) and supports the findings that the distance from the equator affects the age of menarche [19]. There is also an association between AIS prevalence and age at menarche in different geographic latitudes [50,51,52,53,54,55,56,57,58,59,60].

The scoliotic girls in northern countries, with a greater distance from the equator, experience menarche at a later age, which is connected to a higher prevalence of IS in these countries. This concept seems to be true in southern-globe countries as well [61]. This suggests the possible role of geography in the pathogenesis of IS. Unfortunately, since there are no official data about scoliosis prevalence in Serbia and Bosnia and Herzegovina, we were not able to contribute to these findings.

The growth parameters in terms of standing height and weight were also taken into consideration.

Our results showed a significant difference in height between the groups in terms of premenarchal girls in the control group being taller than non-scoliotic girls, as well as postmenarchal girls in the control group being taller than scoliotic girls.

This finding is not in accordance with some previously published results [62,63,64,65,66] and can possibly be explained by the sample size of our control group and the age difference between the groups—that is, the girls in the control group are older (13.63 years of age) compared to scoliotic (12.57 years of age) and non-scoliotic girls (11.51 years of age).

Goldberg et al. [18] found that scoliotic girls are taller when they are younger, but not in adolescence, while Duval-Beaupère did not find any difference in height between scoliotics and healthy children [67].

When analyzing weight according to BMI groups, the majority of participants in all groups had a healthy weight (normal BMI). In the scoliotic group, there were more girls with a low BMI compared to the non-scoliotic and control groups. However, no significant difference in weight according to BMI groups was found (*p* = 0.073). Grivas et al. [64] reported that scoliotic girls from the age of 8 to 12 years are heavier compared to their non-scoliotic counterparts, but they are thinner after the age of 13, while results from another study showed no difference in weight between scoliotics and non-scoliotics [18,62].

A significant difference in the laterality of scoliotic curves in premenarchal and postmenarchal scoliotic girls was found and is in accordance with previously published results [17]. The menarche-positive scoliotic girls showed predominantly right-sided primary curves, while the menarche-negative scoliotic girls had mainly left-sided primary curves. They showed that the primary right curve was dominant in menarche-positive girls in 61%, while the primary left curve was dominant in menarche-negative girls in 64.3%, which is similar to our findings (54.80% in menarche-positive girls and 60.09% in menarche-negative girls).

In menarche-positive girls, the primary right scoliotic curve was dominant in 54.80%, while in menarche-negative girls, the primary left scoliotic curve was dominant in 60.09% (*p* < 0.01), which represents the patterns reflecting developmental theory and scoliosis produced by Goldberg et al. [18] in terms of three developmental gradients: dorso-ventral, cranio-caudal, and left-right. According to the left-right asymmetry, girls who are premenarchal, younger, and less developed present left-sided primary curves, while girls who are postmenarchal, older and more developed, present right-sided primary curves.

The reason for this finding could also be in the similar underlying mechanism of progressive IIS since left curves are dominant both in IIS and premenarchal scoliotic girls. The development of the thorax in progressive IIS is asymmetrical due to a developmental delay of the upper ribs. leading to the funnel-shaped rib cage that seems to derange the symmetric forces of ribs acting on the spine. For some reason, in these children, the thorax does not undergo normal modeling during childhood development, but there is a change in the upper ribs, which do not grow and elevate symmetrically. This can cause spinal deformity due to the inability of the upper rib system to act as a spinal rotation-defending system in the trunk compared to the pelvic rotation-inducing system during gait, particularly when bipedal gait in infants has been established. This hypothesis may also be relevant to the Nottingham concept of IS etiology [68].

The limitations of our study include a relatively small number of girls in the control group (86 participants). In the future, we plan to include in the study more healthy girls in the control group and also to conduct additional analysis according to the age of participants in different groups.

## 6. Conclusions

In Balkan girls from Bosnia and Herzegovina and Serbia, there was no significant difference in the age of menarche between scoliotic and non-scoliotic girls. However, a significant difference was found in the laterality of the primary curve in premenarchal and postmenarchal scoliotic girls. Furthermore, the results showed a significant difference in height between scoliotic and non-scoliotic girls. Further research is needed to put these results in the context of scoliogeny.

## Figures and Tables

**Table 1:** Comparison between scoliotic, non-scoliotic and control groups according to height.

Height (cm)	Scoliotic	Non-Scoliotic	Control	*p* Value
All patients	159.1 ± 12.5	153.8 ± 14.1	166.7 ± 9.7	*p* < 0.001 ^a^
Premenarchal	150.2 ± 12.8	145.2 ± 11.7	154.3 ± 6.0	*p* < 0.001 ^a^
Postmenarchal	165.9 ± 6.7	165.7 ± 6.3	169.8 ± 7.3	*p* < 0.001 ^a^

Results are presented as mean ± sd. ^a^ One-way ANOVA, post hoc Tukey’s HSD.

**Table 2:** Comparison between scoliotic, non-scoliotic and control groups according to BMI.

	Underweight	Healthy Weight	Overweight	Obesity	Severe Obesity	*p* Value
All patients						
Scoliotic	52 (10.5%)	377 (76.3%)	42 (8.5%)	22 (4.5%)	1 (0.2%)	*p* = 0.073 ^a^
Non-scoliotic	32 (6.1%)	404 (77.2%)	58 (11.1%)	26 (5.0%)	3 (0.6%)	
Control	4 (4.7%)	75 (87.2%)	6 (7.0%)	1 (1.2%)	0 (0.0%)	
Premenarchal						
Scoliotic	25 (11.7%)	155 (72.8%)	23 (10.8%)	10 (4.7%)	0 (0.0%)	
Non-scoliotic	24 (7.9%)	226 (74.1%)	36 (11.8%)	18 (5.9%)	1 (0.3%)	*p* = 0.560 ^a^
Control	2 (11.8%)	15 (88.2%)	0 (0.0%)	0 (0.0%)	0 (0.0%)	
Postmenarchal						
Scoliotic	27 (9.6%)	222 (79.0%)	19 (6.8%)	12 (4.3%)	1 (0.4%)	*p* = 0.128 ^a^
Non-scoliotic	8 (3.7%)	178 (81.7%)	22 (10.1%)	8 (3.7%)	2 (0.9%)	
Control	2 (2.9%)	60 (87.0%)	6 (8.7%)	1 (1.4%)	0 (0.0%)	

^a^ Chi-squared test.

**Table 3:** Age and age at the onset of menarche in all three groups of girls: scoliotics, non-scoliotics and healthy girls.

		Menarche			TOTAL
		Yes		No	
Scoliotics	N	281		213	494
	%	56.88%		43.12%	100%
		Age	Age at menarche	Age	Age
	Mean	14.04	12.38	10.63	12.57
	SD	1.50	1.11	1.95	2.40
	Min-max	10–18	9–15	7–16	7–18
Non-scoliotics	N	218		305	523
	%	41.68%		58.32%	100%
		Age	Age at menarche	Age	Age
	Mean	13.92	12.18	9.78	11.51
	SD	1.69	1.22	1.85	2.71
	Min-max	11–18	8–15	7–15	7–18
Control group	N	69		17	86
	%	80.23%		19.77%	100%
		Age	Age at menarche	Age	Age
	Mean	14.07	12.26	11.82	13.63
	SD	1.28	1.22	1.19	1.54
	Min-max	12–17	10–15	10–14	10–17

**Table 4:** Difference in age of menarche in scoliotic and non-scoliotic girls.

	Scoliotic	Non-Scoliotic	Control Group	*p* Value
N	281	218	69	0.168 ^a^
Age (years)	12.38 ± 1.11	12.18 ± 1.22	12.26 ± 1.22	

^a ^*t*-test.

**Table 5:** The mean Cobb angle in postmenarchal and premenarchal scoliotic girls.

	Postmenarchal	Premenarchal
Right	21.36 ± 10.96	19.94 ± 12.29
Lumbar	16.09 ± 5.76	15.00 ± 9.45
Thoracic	23.38 ± 12.16	23.46 ± 13.96
Thoracolumbar	20.67 ± 10.08	15.25 ± 6.88
Left	22.46 ± 11.13	17.39 ± 7.89
Lumbar	25.25 ± 11.92	18.29 ± 9.33
Thoracic	17.59 ± 9.06	16.77 ± 5.46
Thoracolumbar	20.83 ± 9.98	17.04 ± 7.49

**Table 6:** Cobb angle in premenarchal and postmenarchal scoliotic girls according to the laterality of the curve.

Scoliotic	Postmenarchal	Premenarchal	t	*p* Value
Right	21.36 ± 10.96	19.94 ± 12.29	0.930	0.353 ^a^
Left	22.46 ± 11.13	17.39 ± 7.89	4.297	<0.001 ^a^

^a ^*t*-test.

**Table 7:** Distribution of the laterality of primary curves in the scoliotic postmenarchal group.

281 Scoliotic Girls with Menarche			Laterality of Primary Curve		Total
			Left	Right	
Location of Primary Curve	Lumbar	Count	61	23	84
		% Within Location of Primary Curve	72.62%	27.38%	100%
		% of Total	21.71%	8.18%	29.89%
	Thoracic	Count	13	80	93
		% Within Location of Primary Curve	13.98%	86.02%	100%
		% of Total	4.63%	28.47%	33.10%
	Thoraco Lumbar	Count	53	51	104
		% Within Location of Primary Curve	50.96%	49.04%	100%
		% of Total	18.86%	18.15%	37.01%
Total		Count	127	154	281
		% Within Location of Primary Curve	45.20%	54.80%	100%
		% of Total	45.20%	54.80%	100%

**Table 8:** Distribution of the laterality of primary curves in the scoliotic premenarchal group.

213 Scoliotic Girls without Menarche			Laterality of Primary Curve		Total
			Left	Right	
Location of Primary Curve	Lumbar	Count	38	9	47
		% Within Location of Primary Curve	80.85%	19.15%	100%
		% of Total	17.84%	4.22%	22.06%
	Thoracic	Count	14	48	62
		% Within Location of Primary Curve	22.58%	77.42%	100%
		% of Total	6.57%	22.54%	29.11%
	Thoraco Lumbar	Count	76	28	104
		% Within Location of Primary Curve	73.08%	26.92%	100%
		% of Total	35.68%	13.15%	48.83%
Total		Count	128	85	213
		% Within Location of Primary Curve	60.09%	39.91%	100%
		% of Total	60.09%	39.91%	100%

## Data Availability

Data are available on demand.

## Abbreviations

AIS	adolescent idiopathic scoliosis
AP	anteroposterior
BMI	body mass index
CDC	center for control disease and prevention
IS	idiopathic scoliosis
IIS	infantile idiopathic scoliosis
LH	luteinizing hormone
PA	posteroanterior
SRS	Scoliosis Research Society
